# Prospective Associations between Plasma Saturated, Monounsaturated and Polyunsaturated Fatty Acids and Overall and Breast Cancer Risk – Modulation by Antioxidants: A Nested Case-Control Study

**DOI:** 10.1371/journal.pone.0090442

**Published:** 2014-02-27

**Authors:** Camille Pouchieu, Véronique Chajès, François Laporte, Emmanuelle Kesse-Guyot, Pilar Galan, Serge Hercberg, Paule Latino-Martel, Mathilde Touvier

**Affiliations:** 1 Sorbonne Paris Cité, Nutritional Epidemiology Research Team (EREN), Epidemiology and Biostatistics Center, Inserm U1153, Inra U1125, Cnam, University Paris 13, University Paris 5, University Paris 7, Bobigny, France; 2 Nutrition and Metabolism, International Agency for Research on Cancer, Lyon, France; 3 Department of Integrated Biology, University Hospital of Grenoble, Grenoble, France; 4 Public Health Department, Avicenne Hospital, Bobigny, France; Robert Gordon University, United Kingdom

## Abstract

**Background:**

Mechanistic data suggest that different types of fatty acids play a role in carcinogenesis and that antioxidants may modulate this relationship but epidemiologic evidence is lacking. Our aim was to investigate the association between plasma saturated, monounsaturated and polyunsaturated fatty acids (SFAs, MUFAs and PUFAs) and overall and breast cancer risk and to evaluate the potential modulatory effect of an antioxidant supplementation on these relationships.

**Methods:**

A nested case-control study included all first incident cancer cases diagnosed in the SU.VI.MAX study between 1994 and 2002 (n = 250 cases, one matched control/case). Participants to the SU.VI.MAX randomized controlled trial received either vitamin/mineral antioxidants or placebo during this intervention period. Baseline fatty acid composition of plasma total lipids was measured by gas chromatography. Conditional logistic regression was performed overall and stratified by intervention group.

**Results:**

Dihomo-γ-linolenic acid (P_trend_ = 0.002), the dihomo-γ-linolenic/linoleic acids ratio (P_trend_ = 0.001), mead acid (P_trend_ = 0.0004), and palmitoleic acid (P_trend_ = 0.02) were inversely associated with overall cancer risk. The arachidonic/dihomo-γ-linolenic acids ratio (P_trend_ = 0.02) and linoleic acid (P_trend_ = 0.02) were directly associated with overall cancer risk. Similar results were observed for breast cancer specifically. In stratified analyses, associations were only observed in the placebo group. Notably, total PUFAs were directly associated with overall (P_trend_ = 0.02) and breast cancer risk in the placebo group only.

**Conclusion:**

Specific SFAs, MUFAs and PUFAs were prospectively differentially associated with cancer risk. In addition, this study suggests that antioxidants may modulate these associations by counteracting the potential effects of these fatty acids on carcinogenesis.

## Introduction

Mechanistic data suggest that different types of dietary fatty acids may influence carcinogenesis in different ways. For instance, n-3 polyunsaturated fatty acids (PUFAs) may be involved in several mechanisms that counteract carcinogenic processes [1], [2]. In contrast, it has been suggested in rat models that n-3 or n-6 PUFAs may also generate free oxygen radicals and lipid peroxides that convey genotoxic effects [3], [4].

However, epidemiological data remain inconsistent. As estimation of usual dietary fatty acid intake may be prone to measurement errors [5], the use of blood fatty acid biomarkers in epidemiological studies appears as a strategic alternative [6]–[8]. A meta-analysis published in 2004 [9] and including three prospective cohort studies on circulating fatty acids [10]–[12] showed that total n-3 PUFAs were associated with decreased breast cancer risk, while total monounsaturated fatty acids (MUFAs), oleic acid and the saturated palmitic acid were associated with increased breast cancer risk. Since then, several prospective studies have been conducted on circulating fatty acids and the risk of breast cancer [13]–[15], showing contrasting results. Prospective studies have also been published for prostate [16]–[22] and other cancers [19], [23], [24], but results remain overall inconsistent. Thus, new prospective studies are needed.

Moreover, this high level of heterogeneity within epidemiological data may support the existence of other factors that could modulate the relationship between circulating fatty acids and cancer risk, explaining contrasted results across different populations. Mechanistic data from animal models suggest that dietary antioxidants may be good candidates for this modulatory role [25]–[27]. It is possible that the effects of specific fatty acids on cancer risk may be cancelled or even reversed by the presence of antioxidants. So far, a limited number of epidemiologic studies have been published on this topic, and their results were divergent: whereas 2 prospective studies suggested an inverse association of breast cancer risk with combined high intakes of vitamin E and PUFA [28], [29], one prospective study within the French E3N cohort failed to show any significant interaction [30]. In the Alpha-Tocopherol Beta-Carotene Study (ATBC), α-tocopherol supplementation modified the association between serum linoleic acid and prostate cancer risk [31]. Finally, a case-control study reported a decreased risk of breast cancer associated with high arachidonic acid intakes among women with low vitamin E intakes, but an increased risk among women with both high arachidonic acid and vitamin E intakes [32]. To our knowledge, no prospective epidemiologic study has investigated whether the associations between circulating SFAs, MUFAs, PUFAs and overall and breast cancer risk were modified by antioxidant supplementation.

Thus, the objectives of the present study were 1) to prospectively investigate the relationships between plasma SFAs, MUFAs, PUFAs and the risk of overall and breast cancer; and 2) to assess the potential modulatory effect of an antioxidant supplementation on these relationships.

## Materials and Methods

### Study population

The “Supplementation en Vitamines et Minéraux Antioxydants” study (SU.VI.MAX) is a population-based, double-blind, placebo-controlled, randomized trial (Trial Registration clinicaltrials.gov Identifier: NCT00272428) initially designed to assess the effect of a daily antioxidant supplementation on the incidence of cardiovascular disease and cancer [33]. A total of 13,017 subjects were enrolled in 1994–1995. All participants took a single daily capsule of a combination of 120 mg of ascorbic acid, 30 mg of vitamin E, 6 mg of beta carotene, 100 μg of selenium, and 20 mg of zinc, or a placebo. The intervention study lasted 7.5y. Subjects provided written informed consent, and the study was approved by the Ethics Committee for Studies with Human Subjects at the Paris-Cochin Hospital (CCPPRB n°706) and the “Commission Nationale de l'Informatique et des Libertés” (CNIL n°334641).

### Data and blood collection

At enrollment, all participants underwent a clinical examination and anthropometric measurements by the study nurses and physicians. They completed questionnaires regarding sociodemographic data, smoking, physical activity, and medication use. Fasting blood samples were taken up at inclusion from all subjects (before randomization and start of the intervention). Samples were centrifuged immediately after blood draw, and plasma aliquots were then preserved in sodium heparin. Less than one hour after blood draw, plasma aliquots were stored at −20°C in dry ice for shipment to the central biobank (maximum 24 hours), where they were stored frozen in liquid nitrogen (−70°C).

### Case ascertainment

Major health events were self-reported by subjects during follow-up. Investigations were conducted in all declared cancer cases to obtain medical data from participants, physicians, and/or hospitals. All information was reviewed by an independent expert committee and cases were validated by pathological reports and classified using the International Chronic Diseases Classification, 10th Revision, Clinical Modification (ICD-10) [34].

### Nested case-control study

A case-control study nested in the SU.VI.MAX cohort was designed to include all first primary incident cancer cases diagnosed between baseline in 1994 and December 2002. Controls (one per case) were randomly selected among participants with complete follow-up data and without cancer diagnosis by the end of follow-up and were matched by gender, age (±6 months), number of dietary records and intervention group of the trial (antioxidants or placebo).

### Analyses of plasma fatty acid composition

Baseline plasma samples of selected subjects were used to determine the fatty acid composition of total lipids. Lipids were extracted from 150 µl aliquots of plasma with hexane/isopropanol (3∶2, v:v), saponified with NaOH in dry methanol at 100°C, and the fatty acids were methylated with boron trifluoride (14%) in methanol. The fatty acid methyl esters were quantified by gas chromatography using a capillary column (AT-WAX polar 30 m length, 0.25 mm i.d., film thickness 0.25 µm), and hydrogen as carrier gas. Peak identification was made by comparison of their elution times with that of a mixture of commercial standards. Fatty acid composition was expressed as percentages of the total area of all fatty acid peaks. The coefficients of variation (CVs) were <23.8% for the saturated fatty acids (SFAs), <8.0% for cis MUFAs, <12.2% for n-6 PUFAs, <7.7% for n-3 PUFAs and 10.3% for Mead acid.

We calculated the following ratios: total n-3 PUFAs to total n-6 PUFAs, arachidonic acid to dihomo-γ-linolenic acid (indicator of the activity of Δ5-desaturase), dihomo-γ-linolenic acid to linoleic acid (indicator of the activity of Δ6-desaturase and elongase), and oleic acid to stearic acid (indicator of the rate-limiting enzyme Δ9-desaturase).

### Statistical analyses

Baseline characteristics of participants were compared between cancer cases and controls by using conditional logistic regression analyses. ORs and 95% confidence intervals for overall and breast cancer risk associated with quartiles of each plasma fatty acid, fatty acid categories and ratios were examined by using conditional logistic regression models. Multivariate models were adjusted for gender, age, body mass index (BMI), height, intervention group, alcohol intake, physical activity, smoking status, family history of cancer, and educational level. In breast cancer analyses, multivariate models were further adjusted for family history of breast cancer, number of children, menopausal status and use of menopausal hormone therapy at baseline. There was no missing data for covariates except for smoking status, physical activity and educational level for which missing values (less than 5% for each variable) were replaced by the modal value. Adjustment variables were coded as indicated in Table 1. Further adjustments for energy, total lipid, and fruit and vegetable intakes, and number of dietary records (continuous variables) were also tested.

**Table 1:** Baseline characteristics of cancer cases and matched controls.

	Controls		Overall cancer		Breast cancer		p^1^
	(n = 250)		cases (n = 250)		cases (n = 154)		
Gender [n (%)]							
Men	80 (32.0)		80 (32.0)				
Women	170 (68.0)		170 (68.0)				
Age (y)^2^	51.3±6.2		51.0±6.0		49.5±6.0		0.9
BMI [n (%)]							0.04
<25 kg/m^2^	152 (60.8)		166 (66.4)		116 (75.3)		
25 to <30 kg/m^2^	81 (32.4)		57 (22.8)		28 (18.2)		
≥ 30 kg/m^2^	17 (6.8)		27 (10.8)		10 (6.5)		
Height (cm)	165.0±7.8		165.8±7.8		163.1±5.9		0.1
Intervention group [n (%)]							
Yes	115 (46.0)		115 (46.0)		75 (48.7)		
No (placebo)	135 (54.0)		135 (54.0)		79 (51.3)		
Smoking status [n (%)]							0.004
Never smokers	136 (54.4)		121 (48.4)		85 (55.2)		
Former smokers	89 (35.6)		75 (30.0)		36 (23.4)		
Current smokers	25 (10.0)		54 (21.6)		33 (21.4)		
Physical activity [n (%)]							0.1
Low	51 (20.4)		71 (28.4)		49 (31.8)		
Moderate	81 (32.4)		72 (28.8)		48 (31.2)		
High	118 (47.2)		107 (42.8)		57 (37.0)		
Educational level [n (%)]							0.3
Primary	67 (26.8)		54 (21.6)		27 (17.5)		
Secondary	103 (41.2)		104 (41.6)		59 (38.3)		
University	80 (32.0)		92 (36.8)		68 (44.2)		
Alcool intake (g/d)	12.5±16.7		15.5±18.3		9.2±11.1		0.04
Family history^3^ of any cancer (yes. %)	83 (33.2)		89 (35.6)		45 (29.2)		0.6
Family history^3^ of breast cancer (yes. %)^4^	10 (4.0)		25 (10.0)		24 (15.6)		0.001
Menopausal status (yes. %)^4^	70 (28.0)		72 (28.8)		59 (38.3)		0.8
Use of hormonal treatment for menopause (yes. %)^4^	67 (26.8)		71 (28.4)		62 (40.3)		0.6
Number of biologic children^4^	1.9±1.1		1.9±1.2		2.0±1.2		0.6

1 P value for the comparison of overall cancer cases and controls by conditionnal logistic regression.

2 Mean ± SD (all such values).

3 In first or second degree relatives.

4 In women only.

Since cases and controls were matched for the antioxidant supplement group, statistical interaction between antioxidant supplementation and plasma fatty acids could not be formally tested. However, stratified analyses were performed by running the models separately in supplemented and non-supplemented subjects. In sensitivity analyses, models were also performed on the absolute values of fatty acids. All statistical tests were two-sided, and P<0.05 was considered statistically significant, however, we also pointed out results that remained statistically significant with a more conservative threshold (P<0.01). Analyses were performed with SAS software (v9.2; SAS Institute Inc, Cary, North Carolina).

## Results

A total of 250 incident cancer cases were diagnosed during follow-up: 154 breast and 96 other cancer cases (42 prostate, 20 colorectal, 19 lung, and 15 upper aerodigestive tract cancers). In breast cancer cases, 63 were premenopausal and 91 were postmenopausal. 81% were estrogen receptor positive (ER+) and 71% were progesterone receptor positive (PR+). 69% of breast cancers were ductal, 13% were lobular and 18% derived from other histological types. Mean tumor size was 16.2 (±11.75) mm for breast tumor. 250 controls were randomly selected and matched to cases. Median follow-up time was 3.7 y for cancer cases and 7.9 y for controls. The characteristics of overall and breast cancer cases and controls are described in **Table** **1**. Cancer cases were less frequently overweight but more frequently obese, more often current smokers, had higher alcohol intake and had more often family history of breast cancer (for women). Means (±SDs) of the percentages of each plasma fatty acids are shown in **Table** **2** for overall cancer cases, breast cancer cases and controls. In this crude analysis, plasma concentrations of dihomo-γ-linolenic acid, mead acid and the dihomo-γ-linolenic/linoleic acids ratio were lower in cancer cases than in controls. In addition, as expected due to the random design, no difference in baseline plasma fatty acid levels was observed between the placebo and the supplemented group (p>0.05 for all studied fatty acids, data not tabulated).

**Table 2:** Plasma concentrations of fatty acids at baseline among cases and controls.

Fatty acids	Controls		Overall cancer		Breast cancer		P^1^
	(n = 250)		cases (n = 250)		cases (n = 154)		
	*% of total fatty acids (*± *SD)*						
Total SFAs	28.39±2.34		28.05±2.36		27.78±2.14		0.1
14:0 (myristic acid)	1.06±0.44		1.01±0.44		0.97±0.43		0.2
16:0 (palmitic acid)	20.60±1.97		20.32±2.08		20.06±1.97		0.2
18:0 (stearic acid)	6.71±0.77		6.66±0.80		6.69±0.86		0.5
20:0 (arachidic acid)	0.06±0.02		0.06±0.02		0.06±0.02		0.8
Total MUFAs (*cis*)	21.67±3.11		21.58±3.18		21.06±2.73		0.7
16:1 n-7 (palmitoleic acid)	2.23±0.79		2.15±0.84		2.06±0.68		0.3
18:1 n-7 *cis* (vaccenic acid)	1.42±0.24		1.47±0.75		1.49±0.94		0.3
18:1 n-9 (oleic acid)	18.02±2.58		17.95±2.55		17.52±2.21		0.7
Total n-6 PUFAs	44.50±4.57		44.62±4.83		45.38±4.21		0.8
18:2 n-6 (linoleic acid)	33.73±4.70		34.06±4.67		34.83±4.17		0.4
18:3 n-6 (λ-linolenic acid)	0.53±0.22		0.49±0.19		0.45±0.18		0.05
20:2 n-6 (eicosadienoic acid)	0.21±0.05		0.21±0.05		0.21±0.05		0.5
20:3 n-6 (dihomo-γ-linolenic acid)	1.61±0.37		1.52±0.33		1.51±0.36		0.003
20:4 n-6 (arachidonic acid)	8.19±1.53		8.13±1.59		8.18±1.60		0.6
22:4 n-6 (docosatetraenoic acid)	0.2 1±0.09		0.21±0.08		0.20±0.07		0.7
Total n-3 PUFAs	5.09±1.54		5.43±2.50		5.47±2.71		0.08
18:3 n-3 (α-linolenic acid)	0.51±0.15		0.52±0.17		0.52±0.16		0.2
20:5 n-3 (eicosapentaenoic acid)	1.34±0.82		1.52±1.42		1.50±1.55		0.1
22:5 n-3 (docosapentaenoic acid)	0.55±0.14		0.57±0.20		0.57±0.21		0.2
22:6 n-3 (docosahexaenoic acid)	2.69±0.77		2.82±1.05		2.88±1.10		0.1
n-9 PUFAs							
20:3 n-9 (mead acid)	0.15±0.09		0.13±0.05		0.13±0.05		0.01
Total PUFAs	49.58±4.72		50.05±4.67		50.85±3.78		0.2
Ratio							
n-3/n-6	0.12±0.03		0.13±0.08		0.13±0.09		0.09
20:4 n-6/20:3 n-6	5.32±1.58		5.59±1.53		5.70±1.61		0.05
20:3 n-6/18:2n-6	0.05±0.01		0.04±0.01		0.04±0.01		0.004
18:1n-9/18:0	2.73±0.52		2.73±0.49		2.66±0.48		0.9
Quantity of total fatty acids (µmol/L)^2^	11038.23±2013.56		11330.45±2732.49		10870.56±1953.79		0.3

1 P for the comparison of overall cancer cases and controls by unadjusted conditional logistic regression (only matching factors).

2 Mean and SD for total quantity of fatty acids. This information was available for 174 cancer cases (among which 113 breast cancers) and 174 controls).

Associations between plasma fatty acids and overall cancer risk are presented in **Table** **3**. Dihomo-γ-linolenic acid (OR_Q4vs.Q1_ = 0.49 95%CI = 0.28–0.85, *P*_trend_ = 0.002), the dihomo-γ-linolenic/linoleic acids ratio (OR_Q4vs.Q1_ = 0.46, 95%CI = 0.25–0.85, *P*_trend_ = 0.001), mead acid (OR_Q4vs.Q1_ = 0.35 95%CI = 0.19–0.65, *P*_trend_ = 0.0004), and palmitoleic acid (OR_Q4vs.Q1_ = 0.55 95%CI = 0.30–1.01, *P*_trend_ = 0.02) were inversely associated with overall cancer risk. The arachidonic/dihomo-γ-linolenic acids ratio (OR_Q4vs.Q1_ = 1.90, 95%CI = 1.09–3.30, *P*_trend_ = 0.02) and linoleic acid (OR_Q4vs.Q1_ = 1.91, 95%CI = 1.06–3.43, *P*_trend_ = 0.02) were directly associated with overall cancer risk. The associations between mead acid, linoleic and dihomo-γ-linolenic acids and cancer risk persisted after mutual adjustment for each other. Results for dihomo-γ-linolenic acid, mead acid and the dihomo-γ-linolenic/linoleic acids ratio remained statistically significant when a p-value of 0.01 for significance was considered.

**Table 3:** Multivariate conditional logistic regression for the relationship between plasma fatty acid concentrations and overall cancer risk^1^.

Plasma fatty acids	All (n_cases_ = 250 and n_controls_ = 250)					Placebo group (n_cases_ = 135 and n_controls_ = 135)					Intervention group (n_cases_ = 115 and n_controls_ = 115)				
	OR (95%CI)					OR (95%CI)					OR (95%CI)				
	Q1 (ref)	Q2	Q3	Q4	P trend	Q1 (ref)	Q2	Q3	Q4	P trend	Q1 (ref)	Q2	Q3	Q4	P trend
Total SFAs	1	0.96 (0.58–1.61)	0.68 (0.41–1.14)	0.73 (0.44–1.22)	0.1	1	0.79 (0.39–1.63)	0.58 (0.27–1.26)	0.35 (0.16–0.78)	0.01	1	1.22 (0.52–2.86)	0.99 (0.44–2.23)	1.93 (0.85–4.39)	0.2
14:0 (myristic acid)	1	1.27 (0.76–2.10)	0.80 (0.49–1.33)	0.80 (0.47–1.37)	0.2	1	1.13 (0.56–2.27)	0.79 (0.37–1.70)	0.49 (0.22–1.08)	0.05	1	1.75 (0.75–4.09)	0.85 (0.41–1.74)	1.78 (0.76–4.17)	0.6
16:0 (palmitic acid)	1	1.17 (0.70–1.95)	0.63 (0.37–1.07)	0.78 (0.45–1.34)	0.1	1	0.99 (0.48–2.06)	0.65 (0.30–1.40)	0.28 (0.11–0.69)	0.004	1	1.40 (0.59–3.27)	0.68 (0.28–1.63)	2.49 (1.02–6.04)	0.1
18:0 (stearic acid)	1	0.98 (0.59–1.65)	0.78 (0.45–1.35)	1.05 (0.62–1.77)	1.0	1	0.96 (0.45–2.04)	0.38 (0.18–0.84)	0.76 (0.36–1.58)	0.2	1	1.20 (0.53–2.76)	1.65 (0.64–4.25)	2.04 (0.85–4.86)	0.07
20:0 (arachidic acid)	1	0.74 (0.42–1.31)	0.84 (0.48–1.49)	0.83 (0.47–1.46)	0.8	1	0.84 (0.39–1.84)	1.18 (0.52–2.66)	0.91 (0.39–2.11)	1.0	1	0.67 (0.26–1.72)	0.64 (0.28–1.47)	0.84 (0.36–1.95)	0.8
Total MUFAs (cis)	1	1.10 (0.64–1.87)	0.66 (0.38–1.15)	0.97 (0.54–1.71)	0.5	1	1.20 (0.57–2.51)	0.80 (0.36–1.76)	0.91 (0.39–2.12)	0.5	1	1.14 (0.47–2.74)	0.72 (0.30–1.70)	1.26 (0.54–2.95)	0.8
16:1 n-7 (palmitoleic acid)	1	0.91 (0.53–1.56)	0.59 (0.34–1.02)	0.55 (0.30–1.01)	0.02	1	0.48 (0.21–1.13)	0.33 (0.14–0.77)	0.27 (0.11–0.67)	0.004	1	1.91 (0.83–4.40)	1.11 (0.49–2.51)	1.35 (0.52–3.52)	0.8
18:1 n-7 cis (vaccenic acid)	1	0.86 (0.51–1.44)	1.41 (0.84–2.35)	0.88 (0.51–1.51)	0.9	1	0.85 (0.40–1.80)	1.80 (0.85–3.83)	1.16 (0.52–2.58)	0.3	1	0.99 (0.45–2.19)	1.18 (0.55–2.50)	0.65 (0.28–1.51)	0.5
18:1 n-9 (oleic acid)	1	0.98 (0.59–1.65)	0.60 (0.34–1.07)	1.13 (0.63–2.02)	0.8	1	0.87 (0.42–1.81)	0.59 (0.26–1.34)	0.94 (0.41–2.20)	0.7	1	1.28 (0.57–2.88)	0.66 (0.27–1.62)	1.74 (0.71–4.23)	0.6
Total n-6 PUFAs	1	1.20 (0.71–2.04)	1.51 (0.85–2.67)	1.46 (0.85–2.52)	0.1	1	1.43 (0.66–3.06)	2.24 (1.02–4.95)	2.03 (0.90–4.62)	0.06	1	1.10 (0.48–2.55)	1.24 (0.46–3.36)	0.76 (0.32–1.76)	0.6
18:2 n-6 (linoleic acid)	1	1.41 (0.83–2.38)	2.12 (1.20–3.75)	1.91 (1.06–3.43)	0.02	1	1.26 (0.56–2.80)	2.87 (1.21–6.80)	2.26 (0.91–5.62)	0.02	1	2.10 (0.87–5.04)	1.94 (0.82–4.59)	1.09 (0.44–2.74)	0.8
18:3 n-6 (γ-linolenic acid)	1	0.85 (0.49–1.45)	0.98 (0.57–1.70)	0.56 (0.32–0.98)	0.08	1	0.42 (0.19–0.95)	0.46 (0.21–1.01)	0.20 (0.08–0.50)	0.001	1	1.41 (0.58–3.47)	2.27 (0.89–5.79)	1.26 (0.54–2.93)	0.5
20:2 n-6 (eicosadienoic acid)	1	1.23 (0.74–2.04)	1.19 (0.69–2.08)	1.31 (0.77–2.23)	0.4	1	1.03 (0.52–2.03)	1.49 (0.70–3.17)	1.67 (0.78–3.57)	0.1	1	1.46 (0.63–3.36)	0.81 (0.31–2.07)	0.92 (0.40–2.14)	0.6
20:3 n-6 (dihomo-γ-linolenic acid)	1	0.92 (0.54–1.58)	0.52 (0.31–0.88)	0.49 (0.28–0.85)	0.002	1	0.74 (0.34–1.58)	0.47 (0.23–0.96)	0.43 (0.20–0.93)	0.02	1	1.35 (0.57–3.17)	0.54 (0.23–1.26)	0.71 (0.30–1.68)	0.2
20:4 n-6 (arachidonic acid)	1	1.05 (0.62–1.78)	0.92 (0.53–1.59)	0.79 (0.46–1.37)	0.3	1	0.95 (0.47–1.94)	0.95 (0.46–1.97)	0.81 (0.39–1.71)	0.6	1	1.27 (0.53–3.09)	0.78 (0.29–2.07)	0.70 (0.29–1.70)	0.3
22:4 n-6 (docosatetraenoic acid)	1	1.26 (0.72–2.20)	1.00 (0.59–1.69)	0.99 (0.58–1.73)	0.8	1	1.14 (0.56–2.34)	0.76 (0.37–1.57)	1.01 (0.48–2.12)	0.7	1	1.93 (0.70–5.34)	1.43 (0.61–3.35)	1.09 (0.43–2.77)	1.0
Total n-3 PUFAs	1	0.66 (0.38–1.13)	0.90 (0.53–1.54)	0.97 (0.57–1.65)	0.8	1	0.85 (0.40–1.79)	1.02 (0.50–2.08)	1.56 (0.73–2.35)	0.2	1	0.53 (0.22–1.30)	0.77 (0.31–1.90)	0.60 (0.25–1.45)	0.3
18:3 n-3 ( α-linolenic acid)	1	1.27 (0.74–2.20)	1.26 (0.71–2.24)	1.25 (0.73–2.13)	0.5	1	1.10 (0.49–2.45)	1.77 (0.78–4.02)	1.06 (0.50–2.27)	0.8	1	1.36 (0.59–3.13)	0.81 (0.33–1.97)	1.55 (0.64–3.74)	0.6
20:5 n-3 (eicosapentaenoic acid)	1	1.04 (0.63–1.71)	0.54 (0.31–0.93)	1.19 (0.71–2.01)	0.9	1	1.31 (0.64–2.70)	0.41 (0.19–0.88)	1.75 (0.85–3.58)	0.7	1	0.90 (0.41–1.95)	0.63 (0.26–1.57)	0.85 (0.36–2.01)	0.6
22:5 n-3 (docosapentaenoic acid)	1	0.63 (0.38–1.06)	0.70 (0.42–1.16)	0.95 (0.57–1.58)	0.9	1	0.89 (0.45–1.74)	0.63 (0.29–1.34)	1.42 (0.68–2.96)	0.5	1	0.40 (0.16–0.98)	0.65 (0.30–1.41)	0.57 (0.25–1.28)	0.4
22:6 n-3 (docosahexaenoic acid)	1	0.96 (0.57–1.62)	1.13 (0.69–1.84)	1.04 (0.61–1.78)	0.7	1	1.46 (0.68–3.14)	1.32 (0.69–2.52)	1.76 (0.84–3.70)	0.2	1	0.59 (0.26–1.36)	0.86 (0.36–2.02)	0.53 (0.21–1.34)	0.3
n-9 PUFAs															
20:3 n-9 (mead acid)	1	0.66 (0.39–1.13)	0.44 (0.24–0.78)	0.35 (0.19–0.65)	0.0004	1	0.39 (0.18–0.87)	0.34 (0.15–0.78)	0.18 (0.07–0.45)	0.0005	1	1.21 (0.53–2.77)	0.55 (0.21–1.44)	0.86 (0.34–2.16)	0.4
Total PUFAs	1	1.21 (0.70–2.08)	1.34 (0.78–2.28)	1.60 (0.92–2.79)	0.09	1	2.09 (0.95–4.61)	2.79 (1.26–6.21)	2.88 (1.20–6.92)	0.02	1	0.63 (0.26–1.54)	0.61 (0.25–1.48)	0.69 (0.29–1.66)	0.5
Ratios															
n-3/n-6 PUFAs	1	0.91 (0.52–1.58)	0.95 (0.56–1.60)	1.18 (0.67–2.08)	0.5	1	1.11 (0.52–2.37)	1.08 (0.55–2.13)	1.71 (0.80–3.69)	0.2	1	0.78 (0.31–1.98)	0.90 (0.35–2.27)	0.88 (0.34–2.27)	0.9
20:4 n-6/20:3 n-6	1	1.32 (0.78–2.22)	1.65 (0.93–2.92)	1.90 (1.09–3.30)	0.02	1	1.82 (0.92–3.63)	1.99 (0.89–4.45)	1.95 (0.91–4.19)	0.1	1	0.56 (0.22–1.42)	1.01 (0.41–2.50)	1.37 (0.55–3.40)	0.3
20:3 n-6/18:2n-6	1	1.20 (0.68–2.09)	0.60 (0.34–1.05)	0.46 (0.25–0.85)	0.001	1	0.79 (0.34–1.84)	0.43 (0.19–0.98)	0.29 (0.11–0.77)	0.003	1	1.51 (0.66–3.47)	0.77 (0.33–1.83)	0.78 (0.32–1.88)	0.3
18:1n-9/18:0	1	0.90 (0.52–1.55)	0.90 (0.53–1.54)	1.19 (0.69–2.06)	0.6	1	0.94 (0.44–2.01)	0.86 (0.39–1.88)	1.42 (0.64–3.17)	0.4	1	0.94 (0.40–2.20)	1.05 (0.47–2.36)	1.15 (0.50–2.64)	0.7

1 Adjusted for gender, age, intervention group (except in the models stratified on this variable), body mass index, height, smoking status, physical activity, alcohol intake, educational level and family history of cancer.

When analyses were stratified by intervention group (**Table** **3**), no significant association was observed in the antioxidant group. In contrast, in the placebo group, previous significant associations tended to be strengthened, and further associations appeared: total PUFAs were directly associated with overall cancer risk (OR_Q4vs.Q1_ = 2.88, 95%CI = 1.20–6.92, *P*_trend_ = 0.02) whereas γ-linolenic acid (OR_Q4vsQ1_ = 0.20, 95%CI = 0.08–0.50; *P*_trend_ = 0.001), total SFA (OR_Q4vsQ1_ = 0.35 95%CI = 0.16–0.78; *P*_trend_ = 0.01), and palmitic acid (OR_Q4vsQ1_ = 0.28, 95%CI = 0.11–0.29, *P*_trend_ = 0.004) were associated with decreased overall cancer risk. Among these results, those regarding γ-linolenic and palmitic acids remained statistically significant when a p-value of 0.01 for significance was considered.

Similar trends were observed for breast cancer specifically (**Table** **4**) regarding all results (overall and stratified by intervention group) except for findings related to dihomo-γ-linolenic acid that were not statistically significant and for a direct association that was observed between eicosadienoic acid and breast cancer risk in the placebo group (OR_Q4vsQ1_ = 4.10, 95%CI = 0.92–18.39, *P*_trend_ = 0.03). However the later result was no longer statistically significant if a p-value threshold of 0.01 was considered.

**Table 4:** Multivariate conditional logistic regression for the relationship between plasma fatty acid concentrations and breast cancer risk^1^.

Plasma fatty acids	All (n_cases_ = 154 and n_controls_ = 154)					Placebo group (n_cases_ = 79 and n_controls_ = 79)					Intervention group (n_cases_ = 75 and n_controls_ = 75)				
	OR (95%CI)					OR (95%CI)					OR (95%CI)				
	Q1 (ref)	Q2	Q3	Q4	P trend	Q1 (ref)	Q2	Q3	Q4	P trend	Q1 (ref)	Q2	Q3	Q4	P trend
Total SFAs	1	1.40 (0.66–2.99)	0.87 (0.40–1.91)	0.78 (0.38–1.62)	0.4	1	1.80 (0.53–6.13)	0.72 (0.19–2.76)	0.23 (0.06–0.88)	0.02	1	1.02 (0.30–3.44)	0.79 (0.24–2.57)	2.27 (0.75–6.90)	0.2
14:0 (myristic acid)	1	1.49 (0.67–3.33)	0.95 (0.43–2.07)	0.83 (0.36–1.90)	0.4	1	1.32 (0.37–4.62)	0.88 (0.22–3.45)	0.39 (0.10–1.55)	0.1	1	2.19 (0.59–8.13)	1.09 (0.35–3.39)	2.34 (0.64–8.53)	0.3
16:0 (palmitic acid)	1	1.37 (0.63–2.97)	0.49 (0.20–1.21)	0.78 (0.35–1.77)	0.2	1	1.11 (0.33–3.67)	0.45 (0.10–1.96)	0.20 (0.05–0.86)	0.01	1	1.60 (0.49–5.24)	0.46 (0.12–1.74)	2.97 (0.81–10.80)	0.2
18:0 (stearic acid)	1	0.62 (0.28–1.36)	1.15 (0.52–2.55)	1.05 (0.50–2.19)	0.6	1	0.86 (0.23–3.27)	0.49 (0.15–1.66)	0.44 (0.13–1.43)	0.1	1	0.31 (0.09–1.13)	1.76 (0.47–6.68)	2.44 (0.74–8.10)	0.06
20:0 (arachidic acid)	1	1.33 (0.56–3.16)	1.01 (0.43–2.39)	1.36 (0.57–2.39)	0.7	1	1.48 (0.40–5.46)	1.07 (0.27–4.24)	1.21 (0.30–4.87)	0.5	1	1.07 (0.26–4.47)	0.92 (0.24–3.46)	1.94 (0.49–7.71)	0.4
Total MUFAs. cis	1	1.16 (0.52–2.59)	0.34 (0.14–0.86)	0.86 (0.37–2.00)	0.3	1	1.15 (0.33–4.02)	0.23 (0.05–1.04)	0.55 (0.13–2.45)	0.3	1	1.03 (0.31–3.42)	0.41 (0.11–1.62)	1.25 (0.37–4.16)	0.8
16:1 n-7 (palmitoleic acid)	1	0.78 (0.37–1.68)	0.49 (0.21–1.18)	0.41 (0.17–1.01)	0.04	1	0.14 (0.03–0.78)	0.03 (0.00–0.32)	0.03 (0.00–0.29)	0.001	1	1.41 (0.51–3.93)	1.58 (0.47–5.26)	1.48 (0.39–5.62)	0.4
18:1 n-7 cis (vaccenic acid)	1	0.59 (0.27–1.29)	1.33 (0.62–2.86)	0.68 (0.32–1.48)	0.8	1	0.54 (0.14–2.13)	2.57 (0.68–9.74)	0.77 (0.21–2.88)	1.0	1	0.71 (0.25–2.02)	0.99 (0.36–2.75)	0.68 (0.23–1.97)	0.6
18:1 n-9 (oleic acid)	1	0.83 (0.38–1.84)	0.49 (0.20–1.17)	0.95 (0.40–2.27)	0.6	1	0.53 (0.16–1.79)	0.28 (0.07–1.17)	0.46 (0.09–2.23)	0.3	1	1.21 (0.36–4.12)	0.54 (0.14–2.18)	1.72 (0.52–5.74)	0.6
Total n-6 PUFAs	1	0.81 (0.37–1.80)	1.66 (0.75–3.65)	1.42 (0.63–3.24)	0.2	1	0.91 (0.26–3.23)	4.91 (1.07–22.50)	6.77 (1.34–34.18)	0.02	1	0.47 (0.13–1.79)	0.90 (0.26–3.18)	0.35 (0.10–1.25)	0.3
18:2 n-6 (linoleic acid)	1	1.86 (0.88–3.95)	3.78 (1.58–9.04)	2.06 (0.88–4.80)	0.04	1	1.71 (0.51–5.75)	4.83 (1.11–20.97)	6.90 (1.38–34.51)	0.01	1	2.73 (0.82–9.15)	3.26 (0.89–11.89)	0.60 (0.16–2.30)	1.0
18:3 n-6 (γ-linolenic acid)	1	0.95 (0.42–2.14)	0.86 (0.38–1.97)	0.58 (0.25–1.35)	0.2	1	0.28 (0.06–1.19)	0.34 (0.07–1.64)	0.10 (0.02–0.63)	0.07	1	2.43 (0.67–8.77)	1.53 (0.44–5.30)	1.46 (0.44–4.87)	0.9
20:2 n-6 (eicosadienoic acid)	1	0.88 (0.42–1.82)	1.22 (0.53–2.79)	1.13 (0.54–2.36)	0.6	1	0.81 (0.24–2.68)	4.40 (0.95–20.43)	4.10 (0.92–18.39)	0.03	1	1.68 (0.51–5.54)	0.85 (0.24–2.94)	0.70 (0.24–2.06)	0.4
20:3 n-6 (dihomo-γ-linolenic acid)	1	1.55 (0.70–3.42)	0.70 (0.33–1.50)	0.67 (0.31–1.46)	0.1	1	1.32 (0.41–4.28)	0.71 (0.24–2.11)	0.99 (0.29–3.34)	0.8	1	2.61 (0.67–10.26)	0.66 (0.18–2.37)	0.59 (0.18–2.37)	0.2
20:4 n-6 (arachidonic acid)	1	1.12 (0.52–2.43)	1.08 (0.45–2.56)	1.04 (0.44–2.44)	1.0	1	1.02 (0.32–3.27)	1.19 (0.32–4.47)	1.02 (0.26–3.97)	0.9	1	1.35 (0.38–4.86)	0.65 (0.15–2.85)	1.13 (0.32–4.08)	0.8
22:4 n-6 (docosatetraenoic acid)	1	2.44 (1.07–5.56)	1.60 (0.71–3.62)	1.57 (0.72–3.43)	0.5	1	1.29 (0.42–3.96)	1.06 (0.31–3.63)	1.27 (0.40–4.04)	0.8	1	11.21 (2.02–62.08)	2.90 (0.76–6.43)	1.80 (0.50–6.43)	0.7
Total n-3 PUFAs	1	0.62 (0.27–1.43)	0.74 (0.31–1.74)	0.82 (0.36–1.85)	0.9	1	0.70 (0.19–2.57)	0.59 (0.16–2.16)	1.28 (0.32–5.21)	0.7	1	0.41 (0.10–1.69)	1.17 (0.32–4.35)	0.56 (0.18–1.78)	0.5
18:3 n-3 ( α-linolenic acid)	1	1.33 (0.57–3.12)	0.94 (0.38–2.28)	1.08 (0.44–2.62)	0.8	1	0.77 (0.17–3.38)	1.29 (0.31–5.30)	0.73 (0.19–2.77)	1.00	1	1.54 (0.46–2.14)	0.52 (0.13–2.00)	1.43 (0.35–5.81)	1.0
20:5 n-3 (eicosapentaenoic acid)	1	1.12 (0.52–2.40)	0.58 (0.26–1.33)	1.06 (0.48–2.34)	0.7	1	1.58 (0.45–5.48)	0.24 (0.06–0.99)	1.72 (0.44–6.71)	0.9	1	0.88 (0.27–2.87)	0.91 (0.27–3.14)	0.75 (0.22–2.61)	0.6
22:5 n-3 (docosapentaenoic acid)	1	0.93 (0.45–1.93)	0.72 (0.35–1.51)	0.98 (0.45–2.12)	0.8	1	1.41 (0.47–4.20)	0.38 (0.09–1.68)	1.35 (0.34–5.33)	0.8	1	0.57 (0.17–1.91)	0.69 (0.24–1.98)	0.61 (0.20–1.84)	0.3
22:6 n-3 (docosahexaenoic acid)	1	1.14 (0.52–2.51)	0.95 (0.45–2.02)	1.00 (0.52–2.51)	0.9	1	2.70 (0.77–9.41)	1.11 (0.32–3.79)	2.02 (0.59–6.95)	0.5	1	0.64 (0.18–2.29)	0.87 (0.30–2.54)	0.54 (0.17–1.78)	0.3
n-9 PUFAs															
20:3 n-9 (mead acid)	1	0.62 (0.28–1.39)	0.48 (0.20–1.14)	0.35 (0.14–0.90)	0.02	1	0.30 (0.07–1.33)	0.19 (0.04–0.83)	0.02 (0.00–0.23)	0.004	1	0.89 (0.27–2.97)	0.68 (0.18–2.54)	1.11 (0.33–3.72)	1.0
Total PUFAs	1	1.43 (0.63–3.22)	1.40 (0.65–3.03)	1.93 (0.86–4.32)	0.1	1	2.93 (0.66–12.93)	2.89 (0.76–10.94)	6.88 (1.53–30.89)	0.02	1	0.66 (0.18–2.39)	0.69 (0.20–2.33)	0.65 (0.17–2.47)	0.5
Ratios															
n-3/n-6 PUFAs	1	1.13 (0.50–2.58)	0.76 (0.32–1.80)	1.02 (0.45–2.30)	0.8	1	1.30 (0.39–4.39)	0.59 (0.18–1.93)	1.41 (0.42–4.68)	0.9	1	1.01 (0.27–3.74)	1.01 (0.25–4.10)	0.87 (0.25–3.00)	0.7
20:4 n-6/20:3 n-6	1	1.31 (0.59–2.88)	1.32 (0.56–3.10)	2.06 (0.87–4.85)	0.1	1	1.61 (0.52–4.96)	1.61 (0.42–6.14)	1.14 (0.32–3.99)	0.9	1	0.63 (0.13–2.96)	1.24 (0.31–5.04)	3.21 (0.65–16.01)	0.08
20:3 n-6/18:2n-6	1	1.31 (0.55–3.09)	0.67 (0.30–1.49)	0.47 (0.19–1.14)	0.03	1	0.97 (0.23–4.01)	0.43 (0.10–1.83)	0.26 (0.05–1.33)	0.07	1	1.90 (0.56–6.44)	0.88 (0.28–2.72)	0.70 (0.21–2.35)	0.4
18:1n-9/18:0	1	0.55 (0.24–1.27)	0.76 (0.35–1.62)	0.69 (0.31–1.53)	0.5	1	0.61 (0.18–2.05)	0.53 (0.16–1.81)	0.88 (0.24–3.19)	1.0	1	0.30 (0.07–1.24)	1.13 (0.35–3.64)	0.49 (0.15–1.62)	0.8

1 Adjusted for gender, age, intervention group (except in the models stratified on this variable), number of dietary records, body mass index, height, smoking status, physical activity, alcohol intake, educational level, family history of breast cancer, menopausal status, use of hormonal treatment for menopause and number of children.

Further adjustment for energy, total lipid, and fruit and vegetable intakes, and number of dietary records did not modify the findings, neither did the sensitivity analyses excluding cases (n = 30) diagnosed during the first year of follow–up nor excluding the in-situ breast cancer cases (n = 20) (data not shown). Results were also similar when analyses were performed on the absolute values of fatty acids, for subjects with such available data (n = 174 cases and 174 controls) (data not shown).

## Discussion

In this prospective study, we observed inverse associations between dihomo-γ-linolenic acid, the dihomo-γ-linolenic/linoleic acids ratio, γ-linolenic acid (placebo group), mead acid, palmitoleic acid and overall cancer risk, and direct associations between the arachidonic/dihomo-γ-linolenic acids ratio, linoleic acid and overall cancer risk. Similar results were observed for breast cancer specifically. In addition, to our knowledge, this study was the first to prospectively examine the potential modulatory role of an antioxidant supplementation on the relationships between circulating SFAs, MUFAs and PUFAs and overall and breast cancer risk. Interestingly, no association was observed in the antioxidant-supplemented group, whereas all previously described associations were found in the placebo group. Some associations were even observed only in the placebo group, such as a direct association between total PUFAs and overall and breast cancer risk.

We observed inverse associations between dihomo-γ-linolenic acid, the ratio of dihomo-γ-linolenic/linoleic acids (indicator of the Δ6 desaturase and elongase which converts linoleic acid into dihomo-γ-linolenic acid), γ-linolenic acid (placebo group) and overall cancer risk. Consistently, a prospective case-control study nested in the Carotene and Retinol Efficacy Trial (CARET), including 641 cases, reported an inverse association between dihomo-γ-linolenic acid and non-aggressive prostate cancer risk [16]. In contrast, some prospective studies reported direct associations between dihomo-γ-linolenic and gastric adenocarcinoma [23] and prostate cancer [19] risk. Although these associations require further investigation, our findings are supported by mechanistic studies: dihomo-γ-linolenic acid inhibits both motility and invasiveness of human colon cancer cells by increasing the expression of E-cadherin, and it reduces tumor-endothelium adhesion, a key factor in the establishment of distant metastases [35]. Dihomo-γ-linolenic acid interferes in cellular lipid metabolism and eicosanoid (cyclooxygenase and lipoxygenase) biosynthesis. It can be further converted by inflammatory cells to 15-(S)-hydroxy-8,11,13-eicosatrienoic acid and prostaglandin E1 (PGE1), that possess both anti-inflammatory and anti-proliferative properties. PGE1 could also induce growth inhibition and differentiation of cancer cells [35]. Regarding γ-linolenic acid, it has been shown to inhibit the overexpression and hyperactivity of fatty acid synthase oncogene closely linked to malignant transformation of mammary cells [36].

To our knowledge, the inverse association observed in the present study between mead acid and the risk of overall and breast cancer has not been previously documented in epidemiologic studies. Mechanistic data support this result. Mead acid is converted to C3 and D3 leukotrienes, which have an anti-inflammatory effect [37], and opposes 2-series prostaglandin (PGE-2) production from arachidonic acid [38].

Linoleic acid was directly associated with overall and breast cancer risk. Prospective epidemiological studies have generally failed to establish clear evidence of an association between linoleic acid and cancer risk [18], [20], [22], though a significant inverse association has been reported in some studies with breast [9], [13], [15] and prostate [19] cancers. However, our finding on linoleic acid is consistent with animal and in vitro models, which have shown its ability to promote breast and prostate cancer growth [39].

This result is also consistent with the previously discussed inverse association observed between mead acid and cancer risk. Indeed, under normal physiological conditions, n-9 derivatives are formed in small amounts, and a significant increase in mead acid status (a metabolite of oleic acid) suggests a deficiency of n-6 (and n-3) essential fatty acids [40]. Thus, both results are probably interrelated. However, these associations persisted after mutual adjustment for each of these fatty acids.

We found that an increasing concentration of palmitoleic acid was associated with a decreased risk of overall and breast cancer. Consistent with our findings, a case-control study [41] including 291 cancer cases reported a significant reduction in breast cancer risk associated with palmitoleic acid in adipose tissue. In contrast, a meta-analysis conducted in 2004 and involving 3 prospective studies [9] observed direct association between palmitoleic acid and post-menopausal breast cancer risk and one prospective study observed direct association with prostate cancer risk [21]. Cis-palmitoleic acid is mainly found in dairy products, thus, we cannot rule out the fact that its observed association with cancer risk reflects in fact a potentially protective effect of other components of dairy products, such as vitamin D.

The ratio of arachidonic/dihomo-γ-linolenic acids (indicator of the Δ5 desaturase activity) was associated with an increased risk of overall cancer. In line with this finding, a Swedish nested case-control study observed a borderline non-significant increase in breast cancer risk associated with this ratio [14]. This result is supported by mechanistic plausibility: dihomo-γ-linolenic is converted to arachidonic acid that can be converted, via the cyclooxygenase pathway, in PGE-2 that stimulate cancer cell proliferation [35].

One of the most salient and original findings of our study is the fact that antioxidant supplementation strongly modulated the associations between circulating SFAs, MUFAs, PUFAs and cancer risk. In the ATBC Study, serum linoleic acid was inversely associated with prostate cancer risk only among men who received high-dose α-tocopherol supplements (50 mg/day) [31]. In contrast, in our study, no association was found in the intervention group, whereas all previously described associations were observed and generally strengthen in the placebo group. Total PUFAs were associated with increased overall and breast cancer risk in the placebo group, whereas this relationship was not observed in the antioxidant-supplemented group. These results are consistent with mechanisms observed in some experimental studies [4], [26], [27]. Indeed, in addition to the specific and contrasted effects of n-3 and n-6 PUFAs, these studies suggested that when unprotected (low antioxidant status), PUFAs in general could be metabolized and transformed into peroxides that may convey genotoxic effects, whereas antioxidants protect PUFAs from peroxidation, thereby potentially cancelling these carcinogenic properties. In addition, in vitro and in vivo models showed that several PUFAs increased cytotoxic activity of anthracyclines during cancer treatment, but this mechanism was abolished by antioxidant addition (notably α-tocopherol) [42]. The overall PUFA level observed in the present study was similar to the one observed in another French cohort [43] and an Italian study [10]. However, caution is needed in the comparison of circulating fatty acid levels across studies since measurement method may be different [8].

Similarly, the previously discussed inverse association between mead acid and overall and breast cancer risk was observed in the placebo but not in the antioxidant-supplemented group. Indeed, antioxidant supplementation, by preserving essential PUFAs from peroxidation, may limit the synthesis of mead acid.

This modulation by antioxidant intake may explain discrepancies between previous studies investigating the associations between circulating PUFAs and cancer risk [9], [10], [15], [22].

Our results showed an inverse association between SFAs (and more specifically palmitic acid) and the risk of overall and breast cancers in the placebo group only. In contrast, several prospective epidemiological studies have reported direct associations between palmitic acid and prostate [20]–[22] or breast [9] cancer risk, and between total SFAs and breast cancer risk [44]. SFAs can be synthesized endogenously. Palmitic acid is the major fatty acid produced by de novo lipogenesis from acetyl CoA and malonyl CoA and is further desaturated to palmitoleic acid or elongated to stearic acid [45]. Thus, plasma concentrations of SFAs do not systematically reflect SFA intakes but rather endogenous de-novo fatty acid synthesis [46], [47]. Circulating palmitic acid could favour palmitoylation of estrogen β-receptors allowing their tumor suppressor function [48].

Strengths of our study include its prospective design, the wide range of circulating SFAs, MUFAs and PUFAs studied and, for the first time in an epidemiological study, the investigation of a potential modulatory role of an antioxidant supplementation in the association between plasma fatty acids and overall and breast cancer risk.

Some limitations should be acknowledged. First, plasma composition of fatty acids was evaluated only once, at baseline. It would have been interesting to evaluate how this composition varied in time after inclusion, overall and by antioxidant supplementation group, but this information was not available. Indeed, several factors may have modified plasma fatty acids profiles during follow-up, such as variation in endogenous lipogenesis or dietary factors. In addition, it cannot be ruled out that other factors than antioxidant supplementation may also have modified the associations between fatty acid levels and cancer risk during follow-up, such as use of specific drugs or weight change over time. However, these could not be investigated in the present study. Second, the fatty acid composition of plasma was determined based on total lipids. Other biomarkers such as fatty acid composition of plasmatic phospholipids or fatty acids from adipose tissue are more appropriate to reflect long-term fatty acid intake [6]. This limitation could explain why we did not detect some associations. For instance, we observed no relationship between n-3 PUFAs and cancer risk. Another explanation could be that n-3 PUFA intake is too low to exert a protective effect, as suggested in the E3N Study [43]. However, this finding is in agreement with most prospective studies conducted in Western countries [16], [20], [44]. Third, several studies suggested an increasing risk of breast, prostate or colorectal cancer associated with increasing concentrations of some individual *trans*-MUFAs [24], [43], [49], but no information was available for plasma concentrations of *trans* fatty acids in the present study. Fourth, as participants received a combination of antioxidants, it was not possible to identify if one of them was more particularly involved in the modulation of the association between plasma fatty acids and cancer risk. However, it can be postulated that the fat-soluble vitamin E may have played a central role, as previously suggested [31]. Next, regarding the multiple testing, several fatty acids were investigated, thus significant associations occurring purely by chance cannot be excluded. However, we strove to specify our models well, adjusting for the most pertinent covariates to minimize the potential for Type I error. Our initial protocol stipulated an alpha level of 0.05. We did not employ an overly conservative alpha level in order not to decrease the available statistical power and also in order not to increase the likelihood of a Type II error. Our results are hypothesis driven and supported by biologic plausibility, and the number of statistically significant results observed in our study was far above the 5% error of the first kind. Besides, all significant results were observed only in the placebo group, whereas type I error would have led to randomly distributed significant results across supplementation groups. Thus, the observed findings cannot be explained entirely by chance. Next, matching of cases and controls for the supplementation group prevented us from testing the statistical significance of observed interactions between antioxidant supplementation and plasma fatty acids. This should be investigated in future prospective studies. Finally, since the present study is observational and not interventional, causality of observed associations cannot be established. Levels of plasma fatty acids are related to each other. Thus, despite mechanistic plausibility of each observed association, it cannot be ruled out that these relationships may not be causal, but may in fact reflect complex mechanisms that involve interrelated fatty acids.

In conclusion, this prospective study highlighted several inverse or direct associations between specific plasma SFAs, MUFAs, PUFAs and cancer risk that were supported by mechanistic plausibility. Notably, for the first time, we have found a negative association between mead acid and overall and breast cancer risk. Our initial hypothesis of a modulatory effect of an antioxidant supplementation on these relationships was verified. To our knowledge, this had never been investigated before in any epidemiological study on circulating fatty acids and overall or breast cancer risk. Additional prospective studies and mechanistic data are needed to better apprehend the influence of antioxidants on the potential pro- and anti-carcinogenic effects of fatty acids.
